# Feasibility of a behavioral automaticity intervention among African Americans at risk for metabolic syndrome

**DOI:** 10.1186/s12889-019-6675-7

**Published:** 2019-04-16

**Authors:** Heather Fritz, Wassim Tarraf, Aaron Brody, Philip Levy

**Affiliations:** 1 Eugene Applebaum College of Pharmacy and Health Sciences, Occupational Therapy Program, Wayne State University, Detroit, Michigan 48201 USA; 2 Institute of Gerontology, Wayne State University, Detroit, Michigan 48202 USA; 3 Department of Emergency Medicine, Wayne State University, Detroit, Michigan 48201 USA; 4 Department of Emergency Medicine and Assistant Vice President for Translational Science and Clinical Research Innovation, Wayne State University, Detroit, Michigan 48201 USA

**Keywords:** Habits, Behavioral automaticity, Health behavior, Metabolic syndrome, Emergency department

## Abstract

**Background:**

Targeting habit-development (behavioral automaticity) as part of healthy lifestyle behavior change interventions may improve the adoption and maintenance of healthful behaviors. Few studies, however, have evaluated the feasibility of using a habit-development approach to foster the adoption of recommended physical activity and dietary behaviors. We report quantitative and qualitative data from a feasibility study evaluating a habit-formation intervention to foster healthy dietary and physical activity habits among middle aged African Americans with metabolic syndrome.

**Methods:**

Using a non-comparative design we evaluated the feasibility an 8-week, hybrid format (telecoaching and face-to-face sessions), habit-focused intervention targeting the development of healthful dietary and physical activity habit development among 24 African Americans aged 40 and older with metabolic syndrome recruited from the emergency department – a setting where individuals in under-resourced communities often go for primary care. We administered behavioral automaticity measures tailored to participants’ self-selected habits biweekly during the intervention and collected clinical outcomes of systolic blood pressure, weight, waist circumference, and BMI at baseline week 20.

**Results:**

Participant attrition from the program was high (~ 50%). Despite high levels of attrition, 92% of intervention completers were extremely satisfied with the program. Intervention completers also experienced gains in behavioral automaticity for both dietary and physical activity habits. Overall, higher levels of adherence were associated with higher positive gains in automaticity with the statistical significance of the associations being more pronounced for physical activity habit plans relative to dietary habit plans.

**Conclusions:**

Our preliminary data support a habit-development approach for fostering the adoption of healthful dietary and physical activity habits. However, in this pilot study high rates of attrition were seen, suggesting that strategies to improve retention and participant engagement should be included in future studies, particularly when targeting African American emergency department patients.

**Trial registration:**

ClinicalTrials.gov ID: NCT03370419 Registered 12/11/2017, retrospectively registered.

**Electronic supplementary material:**

The online version of this article (10.1186/s12889-019-6675-7) contains supplementary material, which is available to authorized users.

## Background

Together, cardiovascular disease and diabetes cost the U.S. economy a staggering $561.1 billion annually and are the 1st and 7th leading causes of early death among U.S. adults [1]. Metabolic syndrome (MetS) is a cardinal cardiovascular and diabetes risk factor, affecting nearly 35% of U.S. adults, and increasing the risk of developing cardiovascular disease (CVD) and diabetes up to five fold [2, 3]. The diagnostic criteria for MetS include the presence of a large waistline (≥ 40 in. for men and ≥ 35 in. for women) as well as two of the following additional risk factors: blood pressure ≥ 130/85, hemoglobin A1c (HbA1c) of ≥ 5.7%, triglyceride levels ≥150 mg/dL, and HDL cholesterol levels ≤50 mg/dL [2]. While the adverse consequences of MetS overall are substantial, it is particularly impactful on African Americans, especially those who reside in low-resource urban settings [4, 5]. Urban dwelling African Americans experience some of the highest rates of MetS [6] and compared to Caucasians, disparities in prevention and treatment contribute to disproportionate development of CVD related morbidity and mortality [5, 7].

Maintaining a healthy body weight by being physically active (PA) and eating a healthy diet can effectively reduce cardiometabolic risk, even amongst those with MetS. However, few U.S. adults adhere to current PA or dietary recommendations, with lower rates in African Americans than Caucasians [8]. Intervening to assist adoption and maintenance of more healthful behaviors has proven beneficial, yet studies frequently report waning effects once interventions are withdrawn [9].

One promising approach for long-term adherence to lifestyle behavior recommendations is to foster the development of PA and dietary *habits,* defined as behavior patterns operating below conscious awareness that are acquired through context-dependent repetition [10]. Habits develop through frequent context-consistent behavioral performances [11]. Over time, habit–situation associations are incrementally strengthened, which results in highly automatic behaviors that are driven by encountering aspects of the situation (e.g., locations, preceding actions in a sequence, or particular moods) rather than by intentions or motivation [10]. The descriptive literature suggests that much of everyday thought and action is under the control of habits [12], making habit development (HD) a promising target of behavioral interventions.

A handful of studies have translated habit science into lifestyle behavior change interventions [13–16] and the results have been promising. For example, when comparing two weight loss interventions (one with HD components and one without) Carels et al. [13], found that while the total weight lost did not significantly differ between the two approaches, those in the habit-focused arm maintained a greater degree of their lost weight at 6-month post intervention. Lally et al., [14] found that providing participants a combination of advice on habit formation, recommendations for healthful eating behaviors, and self-monitoring checklists resulted in statistically significant reduction in body weight at 8 weeks post intervention when compared to controls. While such preliminary evidence suggests that a HD approach may help individuals adopt and maintain healthful behaviors [10] no studies to date have evaluated the feasibility of a HD among underserved African Americans. African Americans face significant challenges to the development of healthful habits (e.g., lack of resources, unstable life circumstances) making targeted interventions in this population important. A focus on development of behavioral skills and provision of environmental supports necessary for healthful HD that are accessible and acceptable to African Americans with MetS would address these difficulties, potentially improving health outcomes and reducing CVD disparities in the process.

We present the results of pilot feasibility study of an 8-week, theory-based, hybrid, HD intervention designed to increase the strength, operationalized as behavioral automaticity, of PA and dietary habits. Such a feasibility study is essential to shed light on the practicability of our proposed intervention [17] and participant satisfaction with the protocol, as well as to provide initial estimates of screening to enrollment ratios, retention rates, and potential effect sizes needed to successfully evaluate primary and secondary outcomes in a subsequent trial.

## Methods

### Theoretical framework

The Pick two to Stick to (P2S2) HD intervention was guided by a framework synthesized from the theoretical and empirical literature about HD and the information-motivation-behavioral (IMB) model [18]. The synthetic framework addresses the mechanisms that affect the transition of behaviors across the continuum from highly intentional to highly automatic. Important components of HD include having stable frequently occurring opportunities for behavioral performance in contexts that support the development of the new habit. Research about habit also suggests that: (a) simple behaviors are more likely to become habits than more complex behaviors [19]; (b) environments can be purposively modified to promote HD [20]; (c) less-intensive approaches to lifestyle behavior changes may be easier to maintain long term [21]; and gains in habit strength can be detected in as little as two weeks [22]. The IMB model is a validated behavioral change framework that hypothesizes that the prerequisites for behavior change include having condition-specific information about the value of behavior changes, being motivated to change behaviors, and having the requisite behavioral skills to change the target health behavior.

### Study design

The study protocol has been described previously [23]. Briefly, we used a 1-arm, non-comparative design, to evaluate the feasibility and preliminary efficacy of the P2S2 intervention among a sample of 40 African Americans with MetS aged 40 and above who were recruited from the Detroit Receiving Hospital (DRH) emergency department (ED) located in Detroit, MI. The active intervention lasted 8 weeks. Outcome measures were collected at baseline and at week 20. Participants received a total of $75 for completing all study activities. The Wayne State University (WSU) Institutional Review Board approved the study protocol.

### Recruitment and enrollment

English speaking African Americans aged 40 and older who presented to the DRH ED with non-life threatening conditions and who were determined to be safe for discharge home were screened for study eligibility. In Detroit 59% of the population lives in medically underserved areas [24], and subsequent reliance on the ED for primary care is common. While lifestyle behavior counseling is now indicated for adults ages 18 and older to reduce the risk for developing lifestyle related chronic conditions [25]; however, African Americans who reside in under-resourced communities often have reduced access to primary care making lifestyle behavior counseling or referral to related programs for such individuals difficult. In an effort to fill this gap in preventative service delivery, we chose to specifically recruit participants from the ED setting.

As previously described, [23] we chose a modified MetS screening criteria to identify potential participants at the point of care. A diagnosis of MetS is confirmed based on the presences of three of the following five criteria: a large waistline (> 40 in. for men and > 35 in. for women); blood pressure > 130/85 mmHg; HbA1c of 5.7–6.4%; triglyceride levels > 150 mg/dL; and HDL cholesterol levels < 50 mg/dL. However, cholesterol and HbA1c testing were not part of routine care provided by the ED that was part of the study. We therefore used a modified MetS criteria and invited eligible participants to enroll if two or more of the following three cardio-metabolic risk factors were confirmed via point of care testing or were documented in the medical record: waistline ≥40 in. for men and ≥ 35 in. for women; blood pressure ≥ 130/85; and HbA1c of 5.7–6.4%. Exclusion criteria included being pregnant or having a history of a previous diagnosis of resistant hypertension or steroid dependent asthma or emphysema, having a diagnosis of cirrhosis or hepatic failure, having a cardiac event within the last 30 days or being diagnosed with chronic kidney disease on renal replacement therapy or cancer (terminal or undergoing active chemotherapeutic or radiation therapy) [23]. Because we were offering a lifestyle behavior change program, we also excluded individuals taking medications for weight reduction or who were already being involved in a weight reduction program.

### Intervention

#### Interventionist training

To promote fidelity to the manualized P2S2 intervention, interventionist ‘coaches’ completed dedicated training with the study principal investigator (HF), who is a certified health coach, and coaching sessions were administered under her direct supervision. During the intervention, coaching sessions were randomly observed for fidelity to the intervention protocol. For each session, coaches also used session content checklists to document session length, attendance, content, and any deviations from the protocol so that sessions could be rated for protocol fidelity.

#### Intervention structure and content

**Fig. 1:**
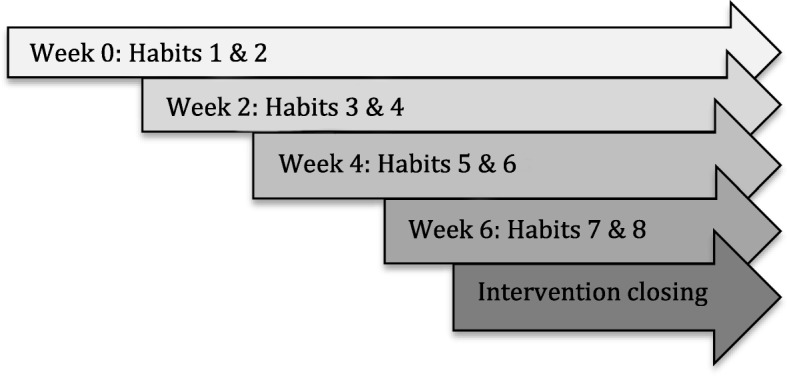
Sequence of habit development

The intervention consisted of five coaching sessions. The intervention began with an initial face-to-face session conducted at the University’s Clinical Research Services Center and that lasted 90–120 min (week 0). The initial session served to build rapport and ensure that all participants were provided the same educational content. Next, participants received four telephone based health-coaching sessions (lasting approximately 30 min) every two weeks (weeks 2, 4, 6, and 8) during which different they set different dietary and physical activity related habit formation goals and created plans to support the development of their selected habits. A follow up data collection visit occurred at the laboratory at week 20. Participants were asked to commit to developing two new habits (one dietary management habit and one PA habit) every 2 weeks over the 8-week intervention period for 8 habits total (see Fig. 1 for the HD sequence). Participants were asked to continue to engage in previous habit plans while also pursuing additional, new plans.

The intervention was manualized to standardized the delivery of the educational content. Content delivered during the initial face-to-face session included an over view of the P2S2 program, as well as educational material about MetS, PA and dietary guidelines, and education about the concept of habit and strategies to promote HD. Based on our previous experience with the target population, educational materials were designed for a 4th–6th grade reading level and used visuals to communicate key ideas. For example, a picture of a first was used to communicate the recommended serving size for proteins and ideas about how to cut down on ‘bad’ fats were communicated using pictures of common food items (e.g., chicken with and without the skin on). Discussions about dietary and exercise topics were also tailored to the local context. For example, we discussed higher fat and lower fat food options at popular restaurants around the city as well as using local resources (e.g., walking trails along the river that were safe and well lit).

The four biweekly telecoaching sessions focused on working with participants to identify low complexity behaviors that could be developed into habits and recurring situations that would eventually serve as the cue for the habitual behavior. For the purposes of the intervention we identified low complexity behaviors as those that.

Participants were instructed to draft habit plan implementation intentions reflecting their selected behavior and situation. In addition, coaches aided participants in identifying environmental modifications that would prompt behavioral performance, as well as reviewing progress towards previous HD goals and trouble shooting as needed. The session structure was intended to allow participants to repeatedly practice applying HD strategies in their own lives with different types of behaviors. See Table 1 for an overview of intervention content.

**Table 1:** Program content and sequence

Session and Format	Content
Session 1: Week 0 Face-to-face	Welcome to the P2S2 program Educational materials: Metabolic syndrome, healthy diet, physical activity, Information and skills training: Principles of HD. HD plan #1 (Habits 1 & 2)
Session 2: Week 2 Telephone	Review educational information (as needed) Review: Assess progress with habits 1 & 2 New: HD plan #2 (Habits 3 & 4)
Session 3: Week 4 Telephone	Review educational information (as needed) Review: Assess progress with habits 1–4 New: HD plan #3 (Habits 5 & 6)
Session 4: Week 6 Telephone	Review educational information (as needed) Review: Assess progress with 1–6 New: HD plan #4 (Habits 7 & 8)
Session 5: Week 8 Telephone	Review: Assess progress with habits 1–8 New: Guidelines for habit maintenance Closure of the program

In addition to coaching sessions, participant received a workbook that included all of the educational materials covered in the face-to-face session as well as worksheets to guide them through constructing and implementing habit-development plans. The workbook also included a tear out self-monitoring log so that participants could track their adherence to their habit formation plans. Participants also received text messages that were specific to their HD plans and that were delivered between one to three times per week (depending on participant preference) at a participant-selected time. Finally, participants were also incentivized through the provision of a welcome bag, which included a meter length of medium resistance exercise band, a pedometer, a USDA My Plate microwave and dishwasher safe portion plate, a pen, and a pad of sticky notes [23].

### Data collection and measures

*Intervention Satisfaction (Measured at week 20)* was assessed using a brief exit interview designed for the purposes of this study. Using a 5 point Likert scale ranging from ‘not satisfied at all’ to ‘extremely satisfied’, the interview queried the following five domains of participant satisfaction: (1) the health coach approach; (2) the frequency and number of contacts; (3) the intervention content; (4) the tailored text messages; and (5) the overall program experience. The interview also included open-ended questions to elicit feedback about the intervention including the number of habits developed over the course of the study and what could be modified to make the program more satisfactory to participants.

#### Behavioral outcomes

The primary study endpoint was self-reported change in behavioral automaticity for participants’ self-selected habits over each of the two-week intervals that corresponded with the initiation of their new HD plans (weeks 0–2; 2–4; 4–6; and 6–8). Behavioral automaticity was assessed using the four-item Self-Reported Behavioral Automaticity Index (SRBAI) [26], which measures self-reported perceptions of behavioral automaticity for an identified behavior. The stem statements are tailored to the needs of the study (e.g., behavior X is something…) and are responded to using the following four response items: “I do automatically”, “I do without having to consciously remember”, “I do without thinking”, and “I start doing before I realize I’m doing it”. The response scale is anchored by agree/disagree. Of 45 reliability assessments of the SRBAI, 23 found α level of .05 within the range. 90–.97, 17 found an alpha between .80–.89, four, an alpha between .70–.79, and one alpha of .68 [26].

Adherence to HD plans across each 14-day measurement period was tracked via participants’ self report of the total number of days that they engaged in their habit plan out of 14 total days possible for each measurement period. Qualitative data including the target behavior that the participant wanted to develop into a habit, the environmental modification to cue the habit, and the desired frequency of text reminders were also collected from participant HD plans.

#### Clinical outcomes (measured at baseline and week 20)

Blood pressure was measured using the BPTru blood pressure device. Weight measurements were taken using a calibrated beam balance scale. Waist circumference was measured using a K-E anthropometric tape in accordance with established anthropometry guidelines [27]. BMI was calculated as weight in kilograms divided by the square of height in meters.

#### Descriptive variables (measured at baseline)

To help characterize the sample for a future trial we collected health literacy data using the seven-item Rapid Estimate of Adult Literacy in Medicine: Short Form [28], comorbid conditions using a comorbidity checklist, and socio-demographic data using as socio-demographic survey designed for the study. A two-item, 5-point Likert scale assessed motivation for making lifestyle changes.

### Data analysis

Qualitative data extracted from participants’ HD plans were analyzed using thematic analysis [29]. Quantitative data analyses were executed in four steps. First, we generated descriptive statistics to compare the characteristics of completers and non-completers. Since this was a feasibility study, our analyses were not intended for inferential purposes; however, we used two-tailed Fisher’s exact test to assess overall differences in categorical variables and t-tests to examine differences in means for continuous variables (Table 2). Additionally, in Additional file 1: Table S1 we present detailed characterization of the time varying outcomes including within, between and overall means and standard deviations. All models focus on bivariate associations and do not control for potential confounders since our sample sizes were prohibitive.

**Table 2:** Sample characteristics by completion status

		Not Completers ^a^		Completers		Fisher’s exact Test
		N	%	N	%	
Gender						
	Male	4	33.3	4	33.3	0.667
	Female	8	66.7	8	66.7	
Relationship Status						
	Never married	3	25.0	5	41.7	
	Married	1	8.3	2	16.7	
	Cohabitating	1	8.3	3	25.0	
	In relationship	0	0.0	1	8.3	
	Separated/divorced	5	41.7	1	8.3	
	Widowed	2	16.7	0	0.0	
Where do you live most of the year						
	Own home	10	83.3	12	100.0	0.478
	Friend/relative	2	16.7	0	0.0	
How many people live with you						0.521
	0	5	41.7	3	25.0	
	1	2	16.7	1	8.3	
	2	3	25.0	1	8.3	
	3	0	0.0	2	16.7	
	4	1	8.3	2	16.7	
	5+	1	8.3	3	25.0	
School						
	8th grade or less	0	0.0	1	8.3	0.183
	Some high school HS)	5	41.7	1	8.3	
	HS or equivalent	2	16.7	5	41.7	
	Some college	5	41.7	5	41.7	
Employment	Full time	5	41.7	0	0.0	0.009
	Part time	1	8.3	5	41.7	
	Unemployed looking for work	0	0.0	3	25.0	
	Unemployed, not looking for work	0	0.0	1	8.3	
	Disabled, not able to work	4	33.3	3	25.0	
	Other	2	16.7	0	0.0	
Income	< $5000	3	27.3	2	16.7	0.510
	$5000–$9999	2	18.2	4	33.3	
	$10,000–$14,999	1	9.1	1	8.3	
	$15,000–$19,999	2	18.2	3	25.0	
	$20,000–$29,999	3	27.3	0	0.0	
	$30,000–$39,999	0	0.0	1	8.3	
	$40,000–$49,000	0	0.0	1	8.3	
		range	Mean	range	Mean	t-test
Health Literacy		0–7	4.4	1–7	5.9	0.11
Age		40–63	51.8	42–61	49.2	0.36
Total motivation score		20–40	34.3	26–40	35.4	0.62
Sum of health conditions		0–7	2.9	0–4	1.6	0.06

^a^Excludes the 16 participants who dropped from the study after initial ED enrollment but prior to attendance at session 1

**Fig. 2:**
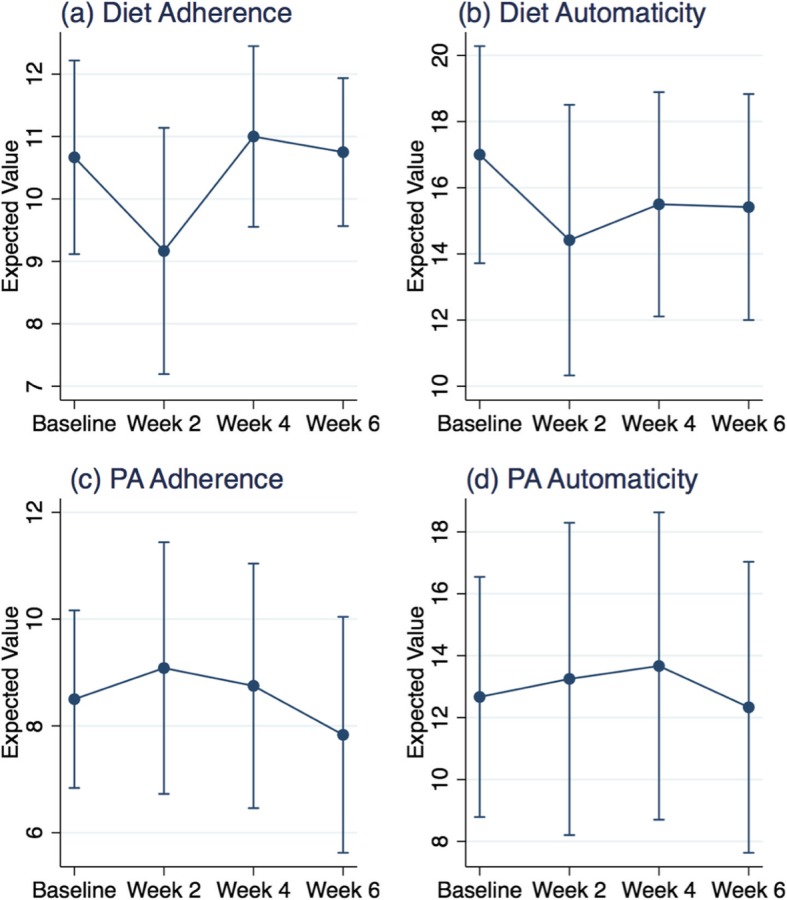
Mean adherence and gains in automaticity by diet and physical activity modalities

Steps 2 through 4 focused on participants who completed the intervention for the duration of the study. In step 2, we estimated the bi-weekly means of diet and PA adherence and calculated their 95% confidence intervals. Mean estimates were based on bivariate generalized estimating equation (GEE) models (outcomes as a function of time) to account for the nested nature of the data (time nested within individuals) assuming a Gaussian distribution for both outcomes and an unstructured covariance matrix. Briefly, GEEs are population average analytic techniques that produce unbiased estimates of regression coefficients while accounting for the dependence among observations (here within individual measurement dependence) as a nuisance modeled through a specified within person correlation structure [30]. GEEs have good properties in small sample sizes, can accommodate multiple outcomes distributions (e.g. binary) and link function, and allow for several correlation structures to be specified a-priori [30, 31]. GEEs have been shown to be robust to “working” covariance misspecification [32]. We used similar modeling procedures to estimate the mean and confidence intervals for the gains in diet and PA automaticity measures. Biweekly gains in automaticity for each modality (diet or PA) were calculated by using differences between pre and post self-reported assessments. The mean estimates and 95% confidence intervals for the adherence and automaticity gains measures by habit modality are plotted in Fig. 2 to facilitate visualization of change in these measures over time.

**Fig. 3:**
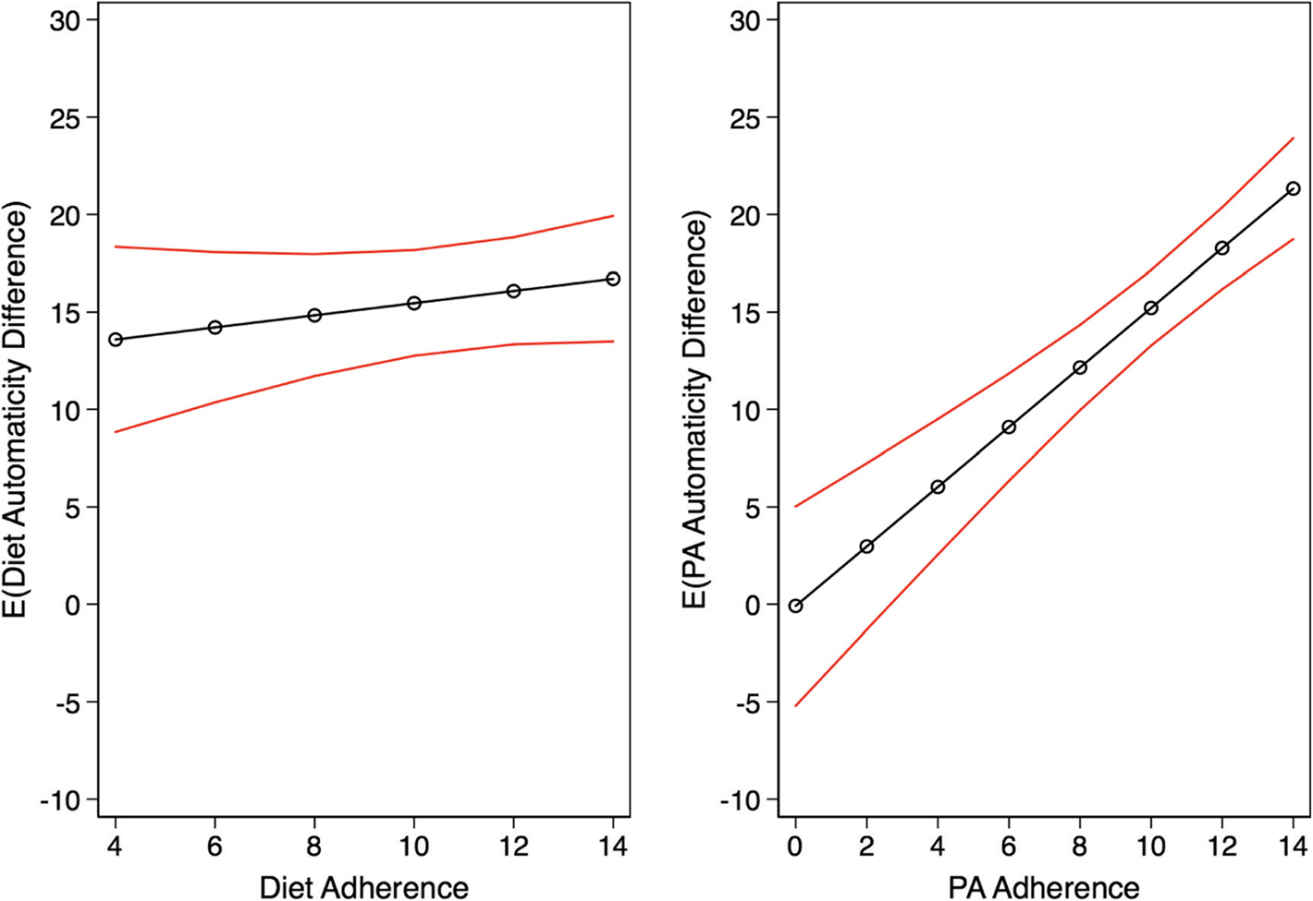
Associations between adherence and gains in automaticity by diet and physical activity modalities

**Fig. 4:**
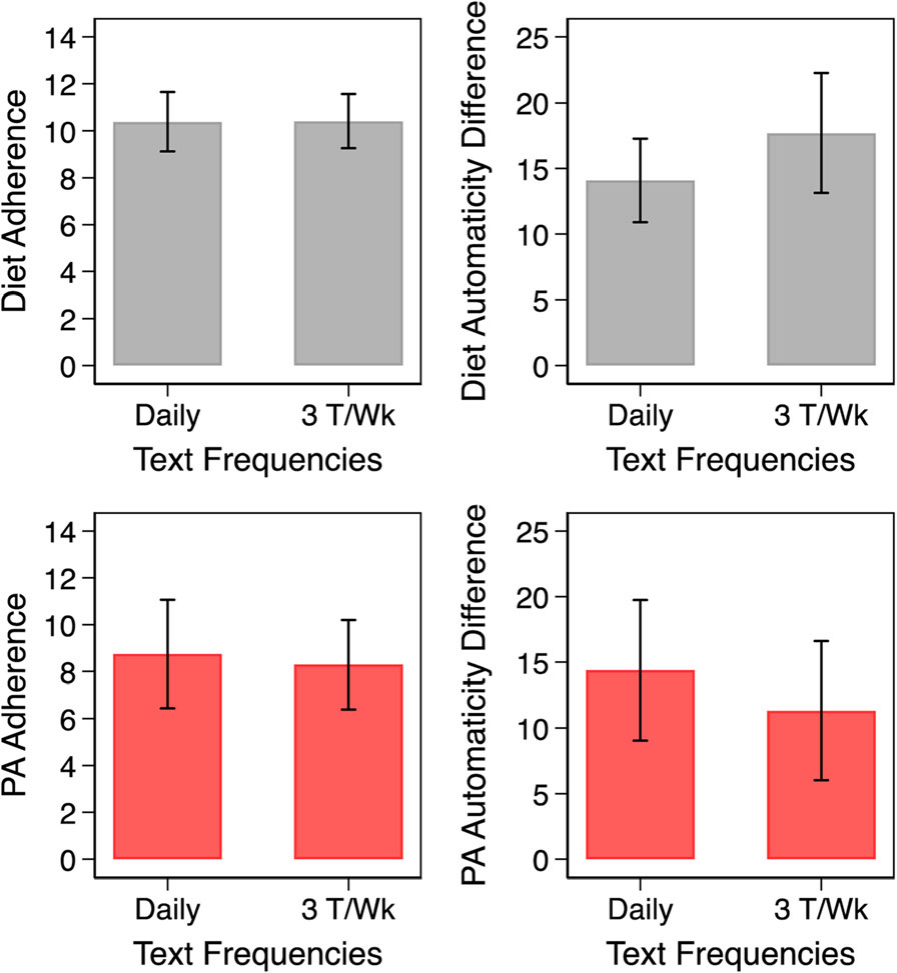
Associations between text frequencies and adherence and gains in automaticity by diet and PA modalitie

In step 3, we used GEEs to examine the associations between adherence and gains in automaticity for each habit modality controlling for measurement time. Subsequently, we estimated and plotted the marginal means of automaticity gains over the continuum of adherence for each modality in Fig. 3. We used similar techniques to examine the associations between texting frequency and both measures of adherence and gains in automaticity for each habit modality also controlling for measurement time. The estimated marginal means of each considered outcome and their 95% confidence intervals are plotted in Fig. 4. Finally, we provide a detailed representation of the generated adherence and automaticity data for both habit modalities and the individual linear fit lines for all completing participants in Additional file 2: Figures S1 and Additional file 3: Figure S2.

### Sensitivity analyses

Recent statistical work on small sample size clustered data indicate that estimates of standard errors can be sensitive to modeling techniques [31, 32]. While GEEs are favored when the primary interest is in population average inferences, other methods (e.g. fixed effects generalized least squares) can lead to more efficient estimates and less prone to be underpowered [32]. However, as a feasibility study we were interested in examining how sensitive the standard errors are to our GEE choice, and whether other methods provide more efficiencies. To do so we reran all models detailed above using 3 alternative recommended techniques: 1) least squares regression with a clustered variance estimator, 2) fixed effects generalized least squares regression, and 3) maximum likelihood mixed effects regression with random intercept. A detailed discussion of the differences in these estimators as well as simulation work, particularly in the context of small sample sizes, have been published recently [32]. The inferences were largely stable with minimal differences in the estimated standard errors. All estimates derived from these models are presented in Additional file 4: Table S2, Additional file 5: Table S3 and Additional file 6: Table S4.

## Results

**Fig. 5:**
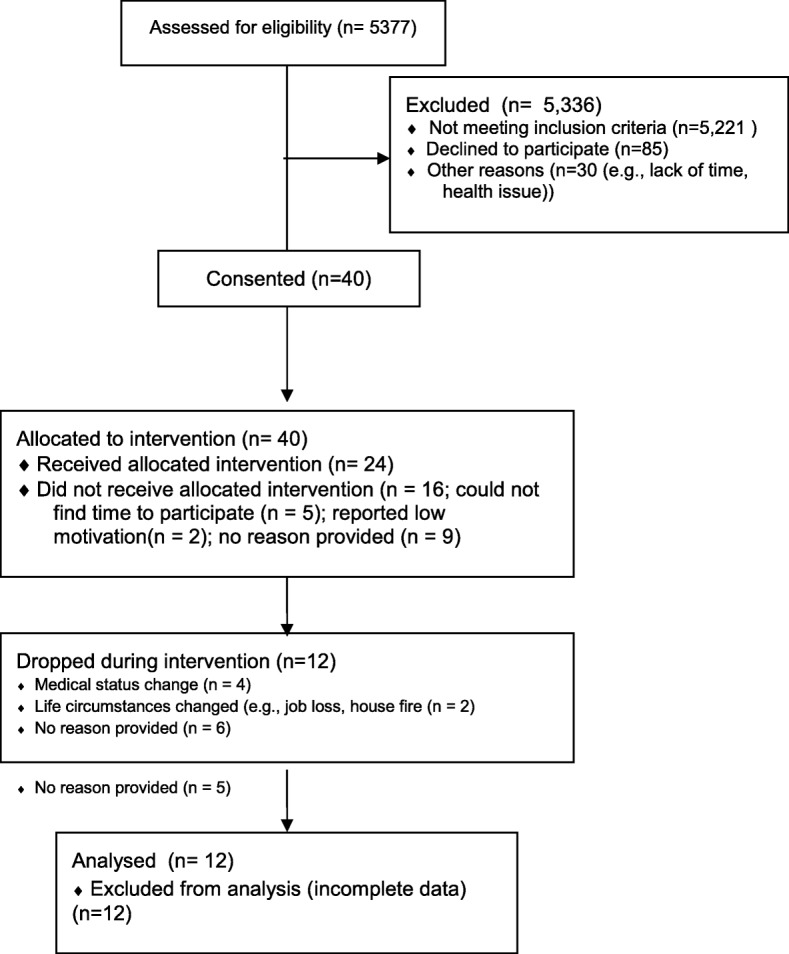
Trial flow

Forty participants were enrolled in P2S2, 16 (40%) of whom dropped from the study between being consented in the ED and the completion of baseline data collection (Fig. 5). Of the 24 participants that completed the baseline data collection visits, 12 completed all intervention visits, six dropped out of the study after the initial coaching session (week 0), five dropped after receiving the second coaching call (week 4), and one participant dropped after the third coaching call (week 6).

Participant characteristics by completion status are presented in Table 2. The average age of completing participants was 49 years, two thirds (67%) were female, a plurality reported never being married (42%), three-fifths lived in households with 3 or more individuals (58.4%), and they unanimously reported living in their own home. Additionally, more than four-fifths of completing participants had more than a high school education (83.4%), 42% reported being unemployed, and 83% reported an income less than $20,000. Average literacy among completers was high (Mean = 5.9; range = 1–7) and so was the average motivation score (Mean = 35.4; range 26–40). The average number of health conditions was 1.6 (range = 0–4). With the exception of employment status (42% of non-completers were employed) and average number of health conditions (2.9 vs. 1.6) there were no substantive differences in characteristics between completers and non-completers.

### Participant satisfaction

Summary statistics for the five different satisfaction measures included in the exit interview indicated that 92% of participants were ‘extremely satisfied’ with the program. The most frequently reported suggested program improvement was to include additional face-to face coaching sessions to help increase accountability and motivation. Participants also suggested increasing the length of time in between HD sessions to decrease burden and to allow more time for prior habits to develop before adding additional habit plans.

### Behavioral outcomes

The average reported biweekly diet and PA adherence scores remained largely unchanged over the study duration. Biweekly gains in diet and PA automaticity also remained constant over the study duration. Average time trends in automaticity gains within each modality were reflective of adherence levels (Fig. 1). Overall, biweekly averages in adherence were consistently higher for diet (M_overall_ = 10.4; SE = 0.4) compared to PA (M_PA-overall_ = 8.5; SE = 0.53). Mean levels of automaticity gains were, with the exception of baseline averages, largely similar across diet and PA behaviors (M_Diet-overall=_15.6; SE = 0.89 vs. M_PA-overall_ = 13.0; SE = 1.15).

Results from the regression analyses examining the association between adherence and automaticity are included in Fig. 5 (also Additional file 5: Table S3). Overall, higher levels of adherence were associated with higher positive gains in automaticity. Both the magnitude and, despite the small sample size, the statistical significance of the association were more pronounced for the PA (β_PA_ = 1.53; SE = 0.24) modality relative to diet (β_Diet_ = 0.31; SE = 0.29).

Finally, we found no evidence to link frequency of texting to adherence outcomes. Frequency of texting, among sampled participants, showed an inverse negative association with diet automaticity gains but a positive association with PA automaticity.

### Habit-development plans

The 12 intervention completers generated *n* = 96 HD plans (*n* = 48 dietary habit plans and *n* = 48 PA plans). Of the 48 dietary habit plans created by participants, *n* = 16 focused on food preparation related habits. Examples included trimming fat off of meat, or using a salt-free seasoning instead of salt on food. Habits related to consuming healthier beverages (*n* = 12), snacks (*n* = 11), and reducing portion size (*n* = 5), were also the focus of HD plans. Four habit plans focused on introducing new foods into the participant’s diet (e.g., using soy milk instead of cows milk on cereal). Environmental modifications to cue behavioral performance included placing the preferred (new) item out in plain view, while hiding away any competing items 43% of the time. Approximately 52% of the time, participant used the study-provided sticky notes as cues (e.g., a note on the bread bag to cue the participant to eat 2 slices instead of 4 slices with dinner). The remaining 5% of the time, participants used phone reminders as the cue.

Walking for 10 min was the most common focus of PA habit plans (*n* = 20 of 48 plans), followed by walking for 30 min (*n* = 7), using the study-provided exercise band or one’s own body weight for resistive exercises (*n* = 15), or engaging in other types of exercise (*n* = 6) (e.g., swimming, working out at a gym, or dancing). Sticky notes and cellular phone reminders were the most common cues that participants used to cue their PA habits (used, 43 and 25% of the time respectively). Durable contextual modifications (e.g., placing exercise equipment in plain view) were only used 32% of the time to cue habits.

## Discussion

As one of the few studies to evaluate the feasibility of delivering a HD intervention, and the only study to our knowledge to have done so among at-risk African Americans recruited from the ED, our findings provide novel insight that is critical for continued engagement with the target population and for understanding the potential utility of a HD approach. Participants reported high degrees of satisfaction with the intervention, yet we experienced higher than expected rates of attrition. Attrition from ED based studies is a known problem, with rates increasing in proportion with the number of post-ED study visits required [33]. Plausible approaches to reduce attrition rates in the future include utilizing home based data collection, increasing the number of face-to-face coaching sessions to build rapport, and the use of a brief run-in period [34] prior to initiating the intervention. Despite the high attrition rates, intervention completers exhibited gains in behavioral automaticity for the majority of their target habits. The data, therefore, contribute to the small, but growing body of translational work suggesting that individuals can at least start to develop new habits through a combination of behavioral intentions and context modifications and that these strategies can work on a range of PA and dietary behaviors.

The P2S2’s biweekly HD sequence was based on a previously conducted HD study [16]. While the authors did not report issues with participants’ pursuing two new habits every two weeks, our participants reported difficulty with the biweekly addition of new HD plans. Though initial gains in HD can be seen in as little as two week, it may take much longer for participants to develop strong habits, [21] especially if life circumstances limit contextual stability (e.g., shift work, changes in child care) or disrupt the frequency and consistency of behavioral engagement [35]. Since the cognitive load reducing benefits of HD would presumably not occur until a ‘strong’ habit was developed, the addition of new HD plans every two weeks may have been overly burdensome to participants.

Another important finding is that some participants struggled to identify more durable environmental modifications to cue engagement in their HD plans and instead relied on sticky notes as cues. It is plausible that such individuals simply could not modify their environments because of others living in the household. It is also plausible that they are not always aware enough of their own daily habits to identify environmental cues to their behaviors [35]. Regardless of the root cause, from a theoretical perspective, using sticky notes or phone alarms could be problematic as once the context changes again (the note is removed) there is no cue to continue to trigger the desired habit.

To our knowledge only one study to date [36] has focused on the qualitative experience of habit formation within the context of a HD intervention approach. The qualitative data presented here, therefore, contribute new insights into the types of behaviors that individuals choose to develop into habits under real world circumstances. Yet it is important to note that we restricted participants to selecting low complexity behaviors that could be engaged in nearly every day and for which they would not have to secure any additional resources or support. We imposed those restrictions based on the descriptive work about habits (e.g., low complexity behaviors are more likely to develop into habits than complex behaviors), and because of sensitivity to the socioeconomic status of our target population we did not want the success or failure of the approach to rest on the participant’s ability to procure additional materials. Nonetheless, the study imposed restrictions likely influences the types of behaviors that individuals chose to develop into habits.

In addition to a small sample size (*n* = 40) and a high rate of attrition, this study is also limited by a short duration of follow-up (12 weeks), and lack of randomization. Therefore, the results should be interpreted with caution until they can be replicated in a larger and more rigorous study. Future research with African Americans with MetS recruited from the ED may facilitate improved tracking of participants through the use of retention strategies tailored to studies recruiting participants from the ED.

## Conclusion

To our knowledge, this study is the first to report on a prospective feasibility pilot of a behavioral automaticity focused lifestyle intervention delivered to a population of medically underserved African Americans with MetS. Although limited by a lack of randomization and short follow-up period, this study provides evidence that a structured behavioral automaticity program can result, at least initially, in habit development. A larger study with a longer follow up period could further elucidate the impact of habit development on clinical outcomes and assess the degree to which habits, once developed, are maintained over time. Lifestyle behavior change interventions targeting underserved African Americans with MetS may help prevent development of cardiovascular and metabolic diseases, while improving overall health.

## Additional files

Additional file 1: **Table S1.** Characterization of the study’s time varying outcomes. (DOCX 82 kb)

Additional file 2: **Figure S1.** Individual linear fit indicating changes in adherence by diet and physical activity modalities for completers. Analysis of changes in adherence by diet and physical activity modalities for completers. (DOCX 186 kb)

Additional file 3: **Figure S2.** Individual linear fit indicating changes in automaticity by diet and physical activity modalities for completers. Analysis of changes in automaticity by diet and physical activity modalities for completers. (DOCX 184 kb)

Additional file 4: **Table S2.** Sensitivity models for examining change in adherence and gains in automaticity across modalities. Analysis of change in adherence and gains in automaticity across modalities. (DOCX 76 kb)

Additional file 5: **Table S3.** Sensitivity models for examining associations between adherence and gains in automaticity across modalities. Associations between adherence and gains in automaticity across modalities. (DOCX 68 kb)

Additional file 6: **Table S4.** Sensitivity models for examining associations between texting frequency, adherence and gains in automaticity across modalities.Description of data: Associations between texting (3 time/week vs., daily) frequency, adherence and gains in automaticity across modalities. (DOCX 77 kb)

## Abbreviations

HD: Habit development
MetS: Metabolic Syndrome
PA: Physical activity

## References

[1] Leading causes of deaths and numbers of deaths, by sex, race, and Hispanic origin United States, 1980 and 2014. Centers for Disease Control, National Center for Health Statistics. 2017. https://www.cdc.gov/nchs/data/hus/hus15.pdf#019.

[2] Health: what is metabolic syndrome. National Heart Lung and Blood Institute. 2011. http://www.nhlbi.nih.gov/health/health-topics/topics/ms

[3] Takahara M, Shimomura I. Metabolic syndrome and lifestyle modification. Rev Endocr Metab Disord. 2014. 10.1007/s11154-014-9294-9298. 10.1007/s11154-014-9294-8 25263290

[4] Graham G (2015). Disparities in cardiovascular disease risk in the United States. Curr Cardiol Rev.

[5] Black/African American profile. U.S. Department of Health and Human Services, Office of Minority Health. 2014. https://www.minorityhealth.hhs.gov/omh/browse.aspx?lvl=3&lvlid=61

[6] Shaya FT, Gu A, Saunders E (2000). Metabolic syndrome prevalence in an urban African American population. Diabetes Metab Syndr Clin Res Rev.

[7] Frieden TR (2013). CDC Health disparities and inequalities report-United States, 2013. Foreword. MMWR Suppl.

[8] American Heart Association, 2017 Statistical Fact Sheet. https://healthmetrics.heart.org/wp-content/uploads/2017/06/2017-Statistical-Fact-Sheet-ucm_492104.pdf

[9] Nilsen WJ, Haverkos L, Nebeling L, Taylor MV (2010). Maintenance of long-term behavior change. Am J Health Behav.

[10] Gardner B. A review and analysis of the use of ‘habit’in understanding, predicting and influencing health-related behaviour. Health Psychol Rev. 2015. 10.1080/17437199.2013.876238. 10.1080/17437199.2013.876238 PMC4566897 25207647

[11] Orbell S, Verplanken B. The automatic component of habit in health behavior: habit as cue-contingent automaticity. Health Psychol. 2010. 10.1037/a0019596. 10.1037/a0019596 20658824

[12] Evans JS. Dual-processing accounts of reasoning, judgment, and social cognition. Annu Rev Psychol. 2008. 10.1146/annurev.psych.59.103006.093629. 10.1146/annurev.psych.59.103006.093629 18154502

[13] Carels RA, Burmeister JM, Koball AM, Oehlhof MW, Hinman N, LeRoy M, Bannon E, Ashrafioun L, Storfer-Isser A, Darby LA, Gumble A. A randomized trial comparing two approaches to weight loss: differences in weight loss maintenance. J Health Psychol. 2014. 10.1177/1359105312470156. 10.1177/1359105312470156 PMC3883879 23349402

[14] Lally P, Chipperfield A, Wardle J. Healthy habits: efficacy of simple advice on weight control based on a habit-formation model. Int J Obes. 2008. 10.1038/sj.ijo.0803771. 10.1038/sj.ijo.0803771 18071344

[15] Mullan B, Allom V, Fayn K, Johnston I. Building habit strength: a pilot intervention designed to improve food-safety behavior. Food Res Int. 2014. 10.1016/j.foodres.2014.09.027.

[16] McGowan L, Cooke LJ, Gardner B, Beeken RJ, Croker H, Wardle J. Healthy feeding habits: efficacy results from a cluster-randomized, controlled exploratory trial of a novel, habit-based intervention with parents. Am J Clin Nutr. 2013. 10.3945/ajcn.112.052159. 10.3945/ajcn.112.052159 23864536

[17] Czajkowski SM, Powell LH, Adler N, Naar-King S, Reynolds KD, Hunter CM, Laraia B, Olster DH, Perna FM, Peterson JC, Epel E. From ideas to efficacy: the ORBIT model for developing behavioral treatments for chronic diseases. Health Psychol. 2015. 10.1037/hea0000161. 10.1037/hea0000161 PMC4522392 25642841

[18] Fisher JD, Fisher WA (1992). Changing AIDS-risk behavior. Psychol Bull.

[19] Wood W, Neal DT. Habit- based behavior change interventions. Behav Sci Policy. 2016; https://behavioralpolicy.org/articles/healthy-through-habit-interventions-for-initiating-maintaining-health-behavior-change/.

[20] Hagobian TA, Phelan S. Lifestyle interventions to reduce obesity and diabetes. Am J Lifestyle Med. 2013. 10.1177/1559827612449600.

[21] Lally P, Van Jaarsveld CH, Potts HW, Wardle J. How are habits formed: modelling habit formation in the real world. Eur J Soc Psychol. 2010. 10.1002/ejsp.674.

[22] Lally P, Gardner B. Promoting habit formation. Health Psychol Rev. 2013. 10.1080/17437199.2011.603640.

[23] Fritz H, Brody A, Levy P (2017). Assessing the feasibility, acceptability, and potential effectiveness of a behavioral-automaticity focused lifestyle intervention for African Americans with metabolic syndrome: the pick two to stick to protocol. Contemp Clin Trials Commun.

[24] Strengthening the safety net in Detroit and Wayne County. Detroit Health Care Stabilization Workgroup. 2003. https://www.michigan.gov/documents/ReportofDetroitHealthCareStabilizationWorkgroup_1_70764_7.pdf

[25] Grossman DC, Bibbins-Domingo K, Curry SJ, Barry MJ, Davidson KW, Doubeni CA, Epling JW, Kemper AR, Krist AH, Kurth AE, Landefeld CS. Behavioral counseling to promote a healthful diet and physical activity for cardiovascular disease prevention in adults without cardiovascular risk factors: US preventive services task force recommendation statement. Jama. 2017. 10.1001/jama.2017.7171. 10.1001/jama.2017.7171 28697260

[26] Gardner B, Abraham C, Lally P, de Bruijn GJ. Towards parsimony in habit measurement: testing the convergent and predictive validity of an automaticity subscale of the self-report habit index. Int J Behav Nutr Phys Act. 2012. 10.1186/1479-5868-9-102. 10.1186/1479-5868-9-102 PMC3552971 22935297

[27] Centers for Disease Control and Prevention (2007). Anthropometry procedures manual.

[28] Arozullah AM, Yarnold PR, Bennett CL, Soltysik RC, Wolf MS, Ferreira RM, Lee SY, Costello S, Shakir A, Denwood C, Bryant FB (2007). Development and validation of a short-form, rapid estimate of adult literacy in medicine. Med Care.

[29] Braun V, Clarke V (2006). Using thematic analysis in psychology. Qualitative Res Psychol.

[30] Hardin JW, Hilbe JM (2013). Generalized estimating equations.

[31] McNeish DM, Harring JR (2017). Clustered data with small sample sizes: comparing the performance of model-based and design-based approaches. Commun Stat Simul Comput.

[32] McNeish D, Stapleton LM (2016). Modeling clustered data with very few clusters. Multivar Behav Res.

[33] Cofield SS, Conwit R, Barsan W, Quinn J (2010). Recruitment and retention of patients into emergency medicine clinical trials. Acad Emerg Med.

[34] Pablos-Méndez A, Barr RG, Shea S (1998). Run-in periods in randomized trials: implications for the application of results in clinical practice. JAMA..

[35] Fritz HA. Learning to do better: the transactional model of diabetes self-management integration. Qual Health Res. 2015. 10.1177/1049732314552453. 10.1177/1049732314552453 PMC5786865 25249549

[36] Fritz H, Cutchin MP. Integrating the science of habit: opportunities for occupational therapy. OTJR. 2016. 10.1177/1539449216643307. 10.1177/1539449216643307 27504882

