# Supplementary material for: Feasibility of a behavioral automaticity intervention among African Americans at risk for metabolic syndrome
Source: BMC Public Health. 2019 Apr 16;19:413. doi: 10.1186/s12889-019-6675-7 (PMC6469067; doi:10.1186/s12889-019-6675-7)
Supplement: Supplementary file 1 — Table S1. Characterization of the study’s time varying outcomes. (DOCX 82 kb) [file 12889_2019_6675_MOESM1_ESM.docx]

| **Variable** |  | **Mean** | **Std. Dev.** | **Min** | **Max** | **Observations** |
| --- | --- | --- | --- | --- | --- | --- |
|  |  |  |  |  |  |  |
| **Diet Automaticity Pre** | overall | 8.48 | 4.58 | 4.00 | 21.00 | N =      48 |
|  | between |  | 3.23 | 4.00 | 13.50 | n =      12 |
|  | within |  | 3.34 |  |  |  |
|  |  |  |  |  |  |  |
| **Diet Automaticity Post** | overall | 24.06 | 4.64 | 11.00 | 28.00 | N =      48 |
|  | between |  | 3.43 | 17.75 | 28.00 | n =      12 |
|  | within |  | 3.24 |  |  |  |
|  |  |  |  |  |  |  |
| **Diet Automaticity (Post-Pre)** | overall | 15.58 | 6.16 | 0.00 | 24.00 | N =      48 |
|  | between |  | 4.88 | 8.25 | 23.50 | n =      12 |
|  | within |  | 3.95 |  |  |  |
|  |  |  |  |  |  |  |
| **PA Automaticity Pre** | overall | 8.10 | 4.74 | 4.00 | 21.00 | N =      48 |
|  | between |  | 2.55 | 4.50 | 13.00 | n =      12 |
|  | within |  | 4.05 |  |  |  |
|  |  |  |  |  |  |  |
| **PA Automaticity Post** | overall | 21.08 | 6.65 | 4.00 | 28.00 | N =      48 |
|  | between |  | 4.68 | 10.50 | 27.00 | n =      12 |
|  | within |  | 4.86 |  |  |  |
|  |  |  |  |  |  |  |
| **PA Automaticity (Post-Pre)** | overall | 12.98 | 8.00 | -8.00 | 24.00 | N =      48 |
|  | between |  | 5.72 | 2.50 | 19.00 | n =      12 |
|  | within |  | 5.78 |  |  |  |
|  |  |  |  |  |  |  |
| **Diet Adherence** | overall | 10.40 | 2.77 | 4.00 | 14.00 | N =      48 |
|  | between |  | 1.55 | 8.25 | 14.00 | n =      12 |
|  | within |  | 2.33 |  |  |  |
|  |  |  |  |  |  |  |
| **PA Adherence** | overall | 8.54 | 3.70 | 0.00 | 14.00 | N =      48 |
|  | between |  | 2.32 | 4.50 | 11.75 | n =      12 |
|  | within |  | 2.94 |  |  |  |
|  |  |  |  |  |  |  |
| **Adherence Difference (Diet -PA)** | overall | 1.85 | 4.69 | -7.00 | 12.00 | N =      48 |
|  | between |  | 2.66 | -3.25 | 6.25 | n =      12 |
|  | within |  | 3.92 |  |  |  |

PA= physical activity. Std. Dev.= Standard deviation. Estimates are based on data from 12 participants with complete data over 4 measurement occasions spanning 8 weeks.
