# Supplementary figures and images for: Feasibility of a behavioral automaticity intervention among African Americans at risk for metabolic syndrome
Source: BMC Public Health. 2019 Apr 16;19:413. doi: 10.1186/s12889-019-6675-7 (PMC6469067; doi:10.1186/s12889-019-6675-7)

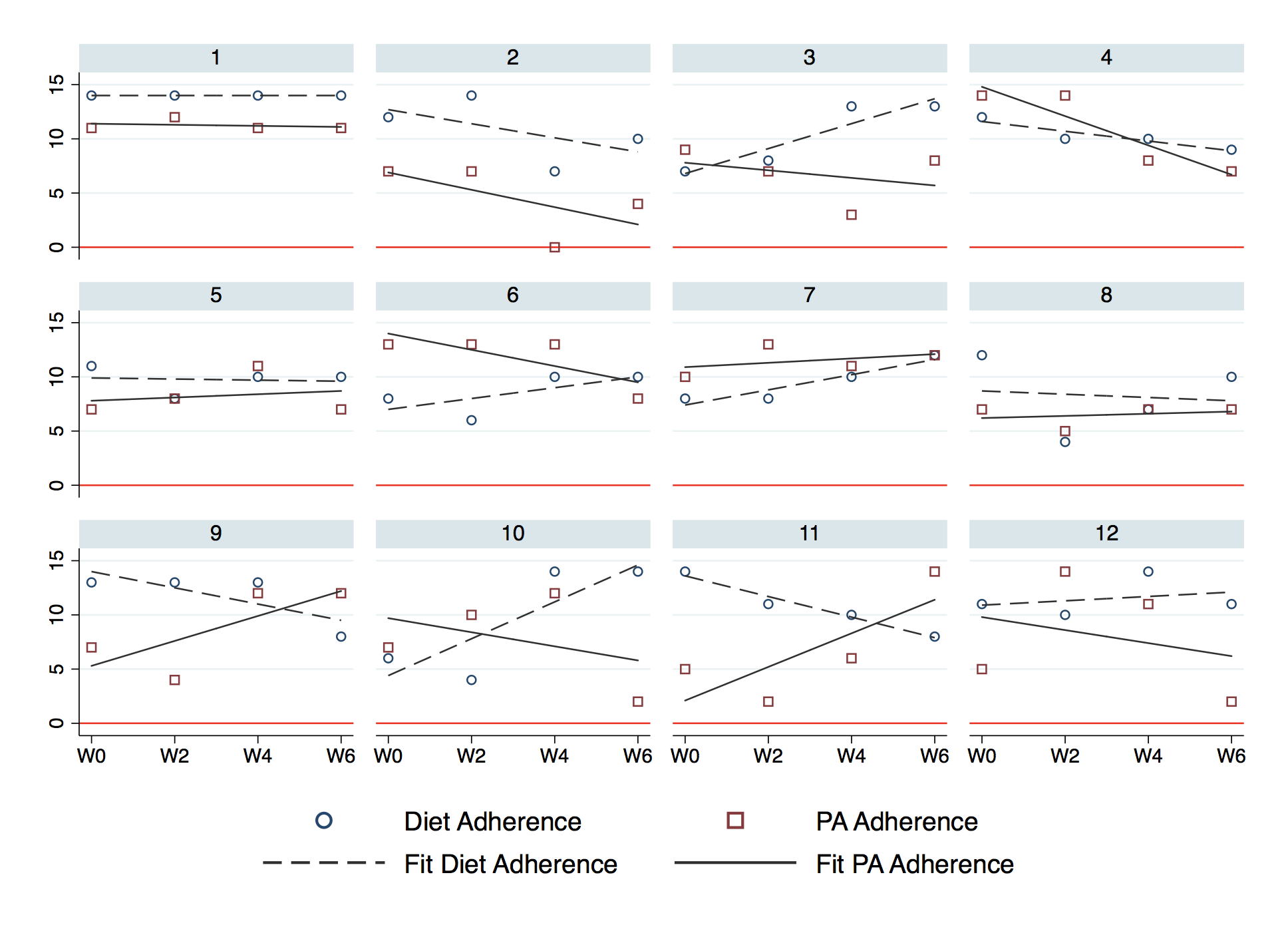

Supplement: Supplementary file 2 — Figure S1. Individual linear fit indicating changes in adherence by diet and physical activity modalities for completers. Analysis of changes in adherence by diet and physical activity modalities for completers. (DOCX 186 kb) [file 12889_2019_6675_MOESM2_ESM.docx]

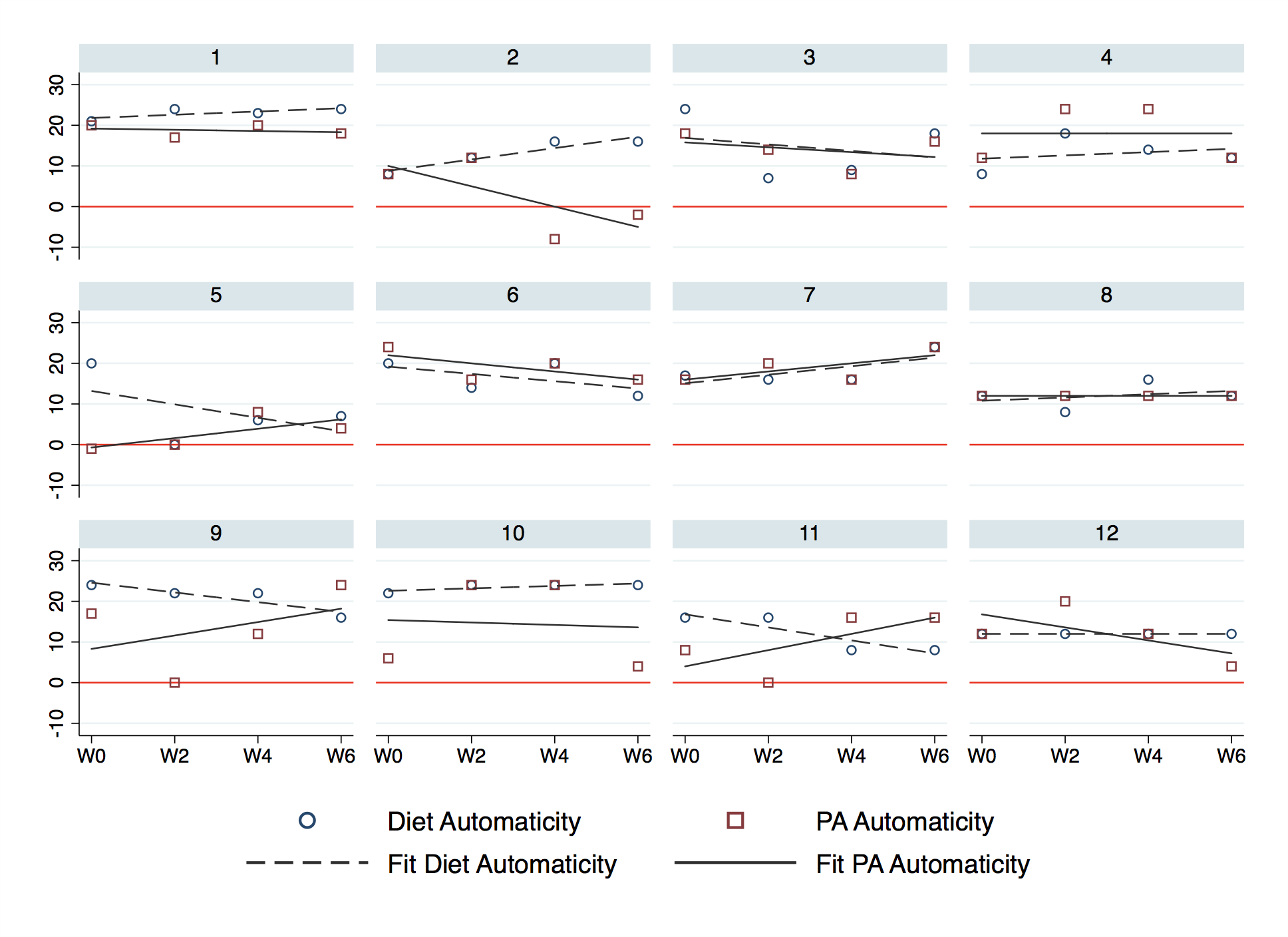

Supplement: Supplementary file 3 — Figure S2. Individual linear fit indicating changes in automaticity by diet and physical activity modalities for completers. Analysis of changes in automaticity by diet and physical activity modalities for completers. (DOCX 184 kb) [file 12889_2019_6675_MOESM3_ESM.docx]
