# Supplementary material for: Feasibility of a behavioral automaticity intervention among African Americans at risk for metabolic syndrome
Source: BMC Public Health. 2019 Apr 16;19:413. doi: 10.1186/s12889-019-6675-7 (PMC6469067; doi:10.1186/s12889-019-6675-7)
Supplement: Supplementary file 4 — Table S2. Sensitivity models for examining change in adherence and gains in automaticity across modalities. Analysis of change in adherence and gains in automaticity across modalities. (DOCX 76 kb) [file 12889_2019_6675_MOESM4_ESM.docx]

| **Diet Adherence** | |  | |  | |  | |  | |
| --- | --- | --- | --- | --- | --- | --- | --- | --- | --- |
|  |  | **Robust OLS** | | **GEE** | | **Fixed Effects Robust GLS** | | **Mixed Model Random Intercept (MLE)** | |
|  |  | b/se | | b/se | | b/se | | b/se | |
|  |  |  | |  | |  | |  | |
|  |  |  | |  | |  | |  | |
|  | Baseline | ref | | ref | | ref | | ref | |
|  |  | n/a | | n/a | | n/a | | n/a | |
|  | Week 2 | -1.5 | | -1.50* | | -1.5 | | -1.5 | |
|  |  | 0.77 | | 0.74 | | 0.77 | | 1.03 | |
|  | Week 4 | 0.33 | | 0.33 | | 0.33 | | 0.33 | |
|  |  | 1.23 | | 1.19 | | 1.23 | | 1.03 | |
|  | Week 6 | 0.08 | | 0.08 | | 0.08 | | 0.08 | |
|  |  | 1.27 | | 1.23 | | 1.27 | | 1.03 | |
|  | Intercept | 10.67*** | | 10.67*** | | 10.67*** | | 10.67*** | |
|  |  | 0.82 | | 0.79 | | 0.68 | | 0.76 | |
|  |  |  | |  | |  | |  | |
|  |  |  | |  | |  | |  | |
|  | Intercept Variance | |  | |  | | 0.61 | |  |
|  |  |  | |  | |  | | 0.98 | |
|  |  |  | |  | |  | |  | |
|  |  |  | |  | |  | |  | |
|  | Residual Variance | |  | |  | | 6.40 | |  |
|  |  |  | |  | |  | | 1.51 | |
|  |  |  | |  | |  | |  | |
|  |  |  | |  | |  | |  | |
| **Physical Activity Adherence** | |  | |  | |  | |  | |
|  |  | **Robust OLS** | | **GEE** | | **Fixed Effects Robust GLS** | | **Mixed Model Random Intercept (MLE)** | |
|  |  | b/se | | b/se | | b/se | | b/se | |
|  |  |  | |  | |  | |  | |
| **Fixed Effects** | |  | |  | |  | |  | |
|  | Baseline | ref | | ref | | ref | | ref | |
|  |  | n/a | | n/a | | n/a | | n/a | |
|  | Week 2 | 0.58 | | 0.58 | | 0.58 | | 0.58 | |
|  |  | 1 | | 0.96 | | 1 | | 1.36 | |
|  | Week 4 | 0.25 | | 0.25 | | 0.25 | | 0.25 | |
|  |  | 1.35 | | 1.3 | | 1.35 | | 1.36 | |
|  | Week 6 | -0.67 | | -0.67 | | -0.67 | | -0.67 | |
|  |  | 1.34 | | 1.3 | | 1.34 | | 1.36 | |
|  | Intercept | 8.50*** | | 8.50*** | | 8.50*** | | 8.50*** | |
|  |  | 0.88 | | 0.85 | | 0.59 | | 1.05 | |
| **Random Effects** | |  | |  | |  | |  | |
|  | Intercept Variance | |  | |  | | 2.18 | |  |
|  |  |  | |  | |  | | 2.12 | |
|  |  |  | |  | |  | |  | |
|  |  |  | |  | |  | |  | |
|  | Residual Variance | |  | |  | | 11.03 | |  |
|  |  |  | |  | |  | | 2.60 | |
|  |  |  | |  | |  | |  | |
|  |  |  | |  | |  | |  | |
| **Diet Automaticity** | |  | |  | |  | |  | |
|  |  | **Robust OLS** | | **GEE** | | **Fixed Effects Robust GLS** | | **Mixed Model Random Intercept (MLE)** | |
|  |  | b/se | | b/se | | b/se | | b/se | |
|  |  |  | |  | |  | |  | |
| **Fixed Effects** | |  | |  | |  | |  | |
|  | Baseline | ref | | ref | | ref | | ref | |
|  |  | n/a | | n/a | | n/a | | n/a | |
|  | Week 2 | -2.58 | | -2.58 | | -2.58 | | -2.58 | |
|  |  | 2.54 | | 2.45 | | 2.54 | | 1.79 | |
|  | Week 4 | -1.5 | | -1.5 | | -1.5 | | -1.5 | |
|  |  | 2.18 | | 2.11 | | 2.18 | | 1.79 | |
|  | Week 6 | -1.58 | | -1.58 | | -1.58 | | -1.58 | |
|  |  | 2.03 | | 1.96 | | 2.03 | | 1.79 | |
|  | Intercept | 17.00*** | | 17.00*** | | 17.00*** | | 17.00*** | |
|  |  | 1.73 | | 1.67 | | 1.56 | | 1.74 | |
| **Random Effects** | |  | |  | |  | |  | |
|  | Intercept Variance |  | |  | |  | | 17.00 | |
|  |  |  | |  | |  | | 8.98 | |
|  |  |  | |  | |  | |  | |
|  |  |  | |  | |  | |  | |
|  | Residual Variance |  | |  | |  | | 19.27 | |
|  |  |  | |  | |  | | 4.54 | |
|  |  |  | |  | |  | |  | |
|  |  |  | |  | |  | |  | |
| **Physical Activity Automaticity** | |  | |  | |  | |  | |
|  |  | **Robust OLS** | | **GEE** | | **Fixed Effects Robust GLS** | | **Mixed Model Random Intercept (MLE)** | |
|  |  | b/se | | b/se | | b/se | | b/se | |
|  |  |  | |  | |  | |  | |
| **Fixed Effects** | |  | |  | |  | |  | |
|  | Baseline | ref | | ref | | ref | | ref | |
|  |  | n/a | | n/a | | n/a | | n/a | |
|  | Week 2 | 0.58 | | 0.58 | | 0.58 | | 0.58 | |
|  |  | 2.85 | | 2.76 | | 2.85 | | 2.69 | |
|  | Week 4 | 1 | | 1 | | 1 | | 1 | |
|  |  | 2.84 | | 2.75 | | 2.84 | | 2.69 | |
|  | Week 6 | -0.33 | | -0.33 | | -0.33 | | -0.33 | |
|  |  | 1.89 | | 1.83 | | 1.89 | | 2.69 | |
|  | Intercept | 12.67*** | | 12.67*** | | 12.67*** | | 12.67*** | |
|  |  | 2.04 | | 1.98 | | 1.34 | | 2.28 | |
| **Random Effects** | |  | |  | |  | |  | |
|  | Intercept Variance | |  | |  | | 19.16 | |  |
|  |  |  | |  | |  | | 12.50 | |
|  |  |  | |  | |  | |  | |
|  |  |  | |  | |  | |  | |
|  | Residual Variance | |  | |  | | 43.26 | |  |
|  |  |  | |  | |  | | 10.20 | |

***p<0.001; *p<0.05

Note: **Betas “b” are fixed effects coefficients from regression models and represent the average magnitude of increase or decrease (based on + or – signs, respectively) in the estimated outcome relative to the reference period (i.e. study baseline). Results** are based on models from (1) Robust ordinary least squares (OLS), (2) Generalized Estimating Equation, (3) Fixed-effects robust generalized least squares (GLS), and Random Intercept mixed effects models using maximum likelihood estimation (MLE) using data from 12 participants with complete data over 4 measurement occasions spanning 8 weeks.
