# Supplementary material for: Feasibility of a behavioral automaticity intervention among African Americans at risk for metabolic syndrome
Source: BMC Public Health. 2019 Apr 16;19:413. doi: 10.1186/s12889-019-6675-7 (PMC6469067; doi:10.1186/s12889-019-6675-7)
Supplement: Supplementary file 5 — Table S3. Sensitivity models for examining associations between adherence and gains in automaticity across modalities. Associations between adherence and gains in automaticity across modalities. (DOCX 68 kb) [file 12889_2019_6675_MOESM5_ESM.docx]

| **Diet Automaticity** | |  |  |  |  |
| --- | --- | --- | --- | --- | --- |
|  |  | **Robust OLS** | **GEE** | **Fixed Effects Robust GLS** | **Mixed Model Random Intercept (MLE)** |
|  |  | b/se | b/se | b/se | b/se |
|  |  |  |  |  |  |
| **Fixed Effects** | |  |  |  |  |
|  | **Diet Adherence** | 0.5 | 0.31 | 0.22 | 0.3 |
|  |  | 0.42 | 0.29 | 0.29 | 0.27 |
| **Time** |  |  |  |  |  |
|  | **Baseline** | Ref | Ref | Ref | Ref |
|  |  | n/a | n/a | n/a | n/a |
|  | **Week 2** | -1.83 | -2.12 | -2.26 | -2.13 |
|  |  | 2.63 | 2.43 | 2.61 | 1.83 |
|  | **Week 4** | -1.67 | -1.6 | -1.57 | -1.6 |
|  |  | 2.47 | 2.28 | 2.32 | 1.78 |
|  | **Week 6** | -1.63 | -1.61 | -1.6 | -1.61 |
|  |  | 2.02 | 1.93 | 2.02 | 1.78 |
|  | **Intercept** | 11.66* | 13.67*** | 14.70*** | 13.77*** |
|  |  | 4.73 | 3.28 | 3.24 | 3.37 |
| **Random Effects** | |  |  |  |  |
|  | Intercept Variance | |  |  | 15.77 |
|  |  |  |  |  | 8.52 |
|  |  |  |  |  |  |
|  |  |  |  |  |  |
|  | Residual Variance | |  |  | 19.02 |
|  |  |  |  |  | 4.49 |
|  |  |  |  |  |  |
| **Physical Activity Automaticity** | |  |  |  |  |
|  |  | **Robust OLS** | **GEE** | **Fixed Effects Robust GLS** | **Mixed Model Random Intercept (MLE)** |
|  |  | b/se | b/se | b/se | b/se |
|  |  |  |  |  |  |
| **Fixed Effects** | |  |  |  |  |
|  | **PA Adherence** | 1.61*** | 1.53*** | 1.41*** | 1.52*** |
|  |  | 0.24 | 0.24 | 0.26 | 0.21 |
| **Time** |  |  |  |  |  |
|  | **Baseline** | Ref | Ref | Ref | Ref |
|  |  | n/a | n/a | n/a | n/a |
|  | **Week 2** | -0.35 | -0.31 | -0.24 | -0.3 |
|  |  | 2.28 | 2.21 | 2.31 | 1.89 |
|  | **Week 4** | 0.6 | 0.62 | 0.65 | 0.62 |
|  |  | 2.77 | 2.64 | 2.7 | 1.89 |
|  | **Week 6** | 0.74 | 0.69 | 0.61 | 0.68 |
|  |  | 1.53 | 1.38 | 1.41 | 1.89 |
|  | **Intercept** | -0.98 | -0.34 | 0.65 | -0.23 |
|  |  | 2.78 | 2.45 | 2.32 | 2.34 |
| **Random Effects** | |  |  |  |  |
|  | Intercept Variance | |  |  | 7.15 |
|  |  |  |  |  | 5.34 |
|  |  |  |  |  |  |
|  |  |  |  |  |  |
|  | Residual Variance | |  |  | 21.35 |
|  |  |  |  |  | 5.06 |

***p<0.001; **p<0.01; *p<0.05

Note: **Betas “b” are fixed effects coefficients from regression models and represent the average magnitude of increase or decrease (based on + or – signs, respectively) in the estimated outcome relative to the reference period (i.e. study baseline).** Results are based on models from (1) Robust ordinary least squares (OLS), (2) Generalized Estimating Equation, (3) Fixed-effects robust generalized least squares (GLS), and Random Intercept mixed effects models using maximum likelihood estimation (MLE) using data from 12 participants with complete data over 4 measurement occasions spanning 8 weeks.
