# Supplementary material for: Feasibility of a behavioral automaticity intervention among African Americans at risk for metabolic syndrome
Source: BMC Public Health. 2019 Apr 16;19:413. doi: 10.1186/s12889-019-6675-7 (PMC6469067; doi:10.1186/s12889-019-6675-7)
Supplement: Supplementary file 6 — Table S4. Sensitivity models for examining associations between texting frequency, adherence and gains in automaticity across modalities.Description of data: Associations between texting (3 time/week vs., daily) frequency, adherence and gains in automaticity across modalities. (DOCX 77 kb) [file 12889_2019_6675_MOESM6_ESM.docx]

| **Adherence Diet** | |  | |  | |  | |  | |  |
| --- | --- | --- | --- | --- | --- | --- | --- | --- | --- | --- |
|  |  | | **Robust OLS** | | **GEE** | | **Fixed Effects Robust GLS** | | **Mixed Model Random Intercept (MLE)** | |
|  |  | | b/se | | b/se | | b/se | | b/se | |
|  |  | |  | |  | |  | |  | |
| **Diet Text** | |  | |  | |  | |  | |  |
|  | Daily | | Ref | | Ref | | Ref | | Ref | |
|  |  | | n/a | | n/a | | n/a | | n/a | |
|  | 3 Text/Week | | 0.03 | | -0.39 | | 1.27 | | 0.16 | |
|  | | 0.58 | | 0.39 | | 1.09 | | 0.84 | |  |
| **Time** |  | |  | |  | |  | |  | |
|  | Baseline | | Ref | | Ref | | Ref | | Ref | |
|  |  | | n/a | | n/a | | n/a | | n/a | |
|  | Week 2 | | -1.5 | | -1.50* | | -1.5 | | -1.5 | |
|  |  | | 0.78 | | 0.75 | | 0.79 | | 1.03 | |
|  | Week 4 | | 0.34 | | 0.3 | | 0.44 | | 0.35 | |
|  |  | | 1.25 | | 1.21 | | 1.21 | | 1.03 | |
|  | Week 6 | | 0.08 | | 0.12 | | -0.02 | | 0.07 | |
|  |  | | 1.29 | | 1.27 | | 1.19 | | 1.03 | |
|  | Intercept | | 10.65*** | | 10.83*** | | 10.14*** | | 10.60*** | |
|  |  | | 0.88 | | 0.76 | | 0.91 | | 0.84 | |
| **Random Effects** | |  | |  | |  | |  | |  |
|  | Intercept Variance | | | |  | |  | | 0.65 | |
|  |  | |  | |  | |  | | 1.02 | |
|  |  | |  | |  | |  | |  | |
|  |  | |  | |  | |  | |  | |
|  | Residual Variance | | | |  | |  | | 6.36 | |
|  |  | |  | |  | |  | | 1.51 | |
|  |  | |  | |  | |  | |  | |
|  |  | |  | |  | |  | |  | |
| **Diet Automaticity** | | | |  | |  | |  | |  |
|  |  | | **Robust OLS** | | **GEE** | | **Fixed Effects Robust GLS** | | **Mixed Model Random Intercept (MLE)** | |
|  |  | | b/se | | b/se | | b/se | | b/se | |
|  |  | |  | |  | |  | |  | |
| **Diet Text** | |  | |  | |  | |  | |  |
|  | Daily | | Ref | | Ref | | Ref | | Ref | |
|  |  | | n/a | | n/a | | n/a | | n/a | |
|  | 3 Text/Week | | 3.6 | | -2.19 | | -0.72 | | 1.62 | |
|  | | 2.29 | | 1.94 | | 1.69 | | 1.91 | |  |
| **Time** |  | |  | |  | |  | |  | |
|  | Baseline | | Ref | | Ref | | Ref | | Ref | |
|  |  | | n/a | | n/a | | n/a | | n/a | |
|  | Week 2 | | -2.58 | | -2.58 | | -2.58 | | -2.58 | |
|  |  | | 2.51 | | 2.52 | | 2.59 | | 1.81 | |
|  | Week 4 | | -1.2 | | -1.68 | | -1.56 | | -1.36 | |
|  |  | | 2.29 | | 2.11 | | 2.22 | | 1.82 | |
|  | Week 6 | | -1.88 | | -1.4 | | -1.52 | | -1.72 | |
|  |  | | 2.06 | | 2.04 | | 2.05 | | 1.82 | |
|  | Intercept | | 15.50*** | | 17.91*** | | 17.30*** | | 16.32*** | |
|  |  | | 1.71 | | 1.78 | | 1.89 | | 1.86 | |
| **Random Effects** | |  | |  | |  | |  | |  |
|  | Intercept Variance | | | |  | |  | | 14.37 | |
|  |  | |  | |  | |  | | 8.45 | |
|  |  | |  | |  | |  | |  | |
|  |  | |  | |  | |  | |  | |
|  | Residual Variance | | | |  | |  | | 19.73 | |
|  |  | |  | |  | |  | | 4.73 | |
|  |  | |  | |  | |  | |  | |
|  |  | |  | |  | |  | |  | |
|  |  | |  | |  | |  | |  | |
|  |  | |  | |  | |  | |  | |
| **Physical Activity (PA) Adherence** | |  | |  | |  | |  | |  |
|  |  | | **Robust OLS** | | **GEE** | | **Fixed Effects Robust GLS** | | **Mixed Model Random Intercept (MLE)** | |
|  |  | | b/se | | b/se | | b/se | | b/se | |
|  |  | |  | |  | |  | |  | |
| **PA Text** | |  | |  | |  | |  | |  |
|  | Daily | | Ref | | Ref | | Ref | | Ref | |
|  |  | | n/a | | n/a | | n/a | | n/a | |
|  | 3 Text/Week | | -0.46 | | -0.13 | | -3.19 | | -1.04 | |
|  | | 1.33 | | 0.99 | | 1.47 | | 1.21 | |  |
| **Time** |  | |  | |  | |  | |  | |
|  | Baseline | | Ref | | Ref | | Ref | | Ref | |
|  |  | | n/a | | n/a | | n/a | | n/a | |
|  | Week 2 | | 0.55 | | 0.57 | | 0.32 | | 0.5 | |
|  |  | | 1.04 | | 0.99 | | 1.04 | | 1.33 | |
|  | Week 4 | | 0.17 | | 0.23 | | -0.28 | | 0.08 | |
|  |  | | 1.33 | | 1.3 | | 1.38 | | 1.34 | |
|  | Week 6 | | -0.63 | | -0.66 | | -0.4 | | -0.58 | |
|  |  | | 1.35 | | 1.28 | | 1.37 | | 1.33 | |
|  | Intercept | | 8.73*** | | 8.57*** | | 10.09*** | | 9.02*** | |
|  |  | | 1.21 | | 1.07 | | 1.07 | | 1.21 | |
| **Random Effects** | |  | |  | |  | |  | |  |
|  | Intercept Variance | | | |  | |  | | 2.69 | |
|  |  | |  | |  | |  | | 2.41 | |
|  |  | |  | |  | |  | |  | |
|  |  | |  | |  | |  | |  | |
|  | Residual Variance | | | |  | |  | | 10.55 | |
|  |  | |  | |  | |  | | 2.53 | |
|  |  | |  | |  | |  | |  | |
|  |  | |  | |  | |  | |  | |
| **PA Automaticity** | | | |  | |  | |  | |  |
|  |  | | **Robust OLS** | | **GEE** | | **Fixed Effects Robust GLS** | | **Mixed Model Random Intercept (MLE)** | |
|  |  | | b/se | | b/se | | b/se | | b/se | |
|  |  | |  | |  | |  | |  | |
| **PA Text** | |  | |  | |  | |  | |  |
|  | Daily | | Ref | | Ref | | Ref | | Ref | |
|  |  | | n/a | | n/a | | n/a | | n/a | |
|  | 3 Text/Week | | -3.07 | | -6.91* | | -6.64 | | -4.34 | |
|  | | 3.41 | | 2.88 | | 4.72 | | 2.62 | |  |
| **Time** |  | |  | |  | |  | |  | |
|  | Baseline | | Ref | | Ref | | Ref | | Ref | |
|  |  | | n/a | | n/a | | n/a | | n/a | |
|  | Week 2 | | 0.33 | | 0.01 | | 0.03 | | 0.22 | |
|  |  | | 2.75 | | 2.48 | | 2.56 | | 2.59 | |
|  | Week 4 | | 0.49 | | -0.15 | | -0.11 | | 0.28 | |
|  |  | | 2.39 | | 2.27 | | 2.56 | | 2.62 | |
|  | Week 6 | | -0.08 | | 0 | | 0.22 | | 0.03 | |
|  |  | | 1.94 | | . | | 2 | | 2.59 | |
|  | Intercept | | 14.20*** | | 16.12*** | | 15.98*** | | 14.84*** | |
|  |  | | 2.22 | | 1.97 | | 2.31 | | 2.6 | |
| **Random Effects** | |  | |  | |  | |  | |  |
|  | Intercept Variance | | | |  | |  | | 20.61 | |
|  |  | |  | |  | |  | | 12.99 | |
|  |  | |  | |  | |  | |  | |
|  |  | |  | |  | |  | |  | |
|  | Residual Variance | | | |  | |  | | 39.95 | |
|  |  | |  | |  | |  | | 9.47 | |

***p<0.001; **p<0.01; *p<0.05

Note: Results are based on models from (1) Robust ordinary least squares (OLS), (2) Generalized

Estimating Equation, (3) Fixed-effects robust generalized least squares (GLS), and Random Intercept mixed effects models using maximum likelihood estimation (MLE) using data from 12 participants with complete data over 4 measurement occasions spanning 8 weeks.
